# Metal Ion Binding in Wild-Type and Mutated Frataxin: A Stability Study

**DOI:** 10.3389/fmolb.2022.878017

**Published:** 2022-05-31

**Authors:** S. Morante, S. Botticelli, R. Chiaraluce, V. Consalvi, G. La Penna, L. Novak, A. Pasquo, M. Petrosino, O. Proux, G. Rossi, G. Salina, F. Stellato

**Affiliations:** ^1^ Dipartimento di Fisica, Universitá di Roma Tor Vergata, Rome, Italy; ^2^ INFN, Sezione di Roma Tor Vergata, Rome, Italy; ^3^ Dipartimento di Scienze Biochimiche “A. Rossi Fanelli”, Sapienza Universitá di Roma, Rome, Italy; ^4^ CNR—Istituto di Chimica dei Composti Organometallici, Firenze, Italy; ^5^ ENEA CR Frascati, Diagnostics and Metrology Laboratory FSN-TECFIS-DIM, Frascati, Italy; ^6^ Chair of Pharmacology, Section of Medicine, University of Fribourg, Fribourg, Switzerland; ^7^ Observatoire des Sciences de L’Univers de Grenoble, UAR 832 CNRS, Université Grenoble Alpes, Grenoble, France; ^8^ Museo Storico della Fisica e Centro Studi e Ricerche E. Fermi, Roma, Italy

**Keywords:** frataxin, metal ions (Co^2+^), frataxin mutants, XAS (XAFS, XANES), termal stability

## Abstract

This work studies the stability of wild-type frataxin and some of its variants found in cancer tissues upon Co^2+^ binding. Although the physiologically involved metal ion in the frataxin enzymatic activity is Fe^2+^, as it is customarily done, Co^2+^ is most often used in experiments because Fe^2+^ is extremely unstable owing to the fast oxidation reaction Fe^2+^ → Fe^3+^. Protein stability is monitored following the conformational changes induced by Co^2+^ binding as measured by circular dichroism, fluorescence spectroscopy, and melting temperature measurements. The stability ranking among the wild-type frataxin and its variants obtained in this way is confirmed by a detailed comparative analysis of the XAS spectra of the metal-protein complex at the Co K-edge. In particular, a fit to the EXAFS region of the spectrum allows positively identifying the frataxin acidic ridge as the most likely location of the metal-binding sites. Furthermore, we can explain the surprising feature emerging from a detailed analysis of the XANES region of the spectrum, showing that the longer 81-210 frataxin fragment has a smaller propensity for Co^2+^ binding than the shorter 90-210 one. This fact is explained by the peculiar role of the N-terminal disordered tail in modulating the protein ability to interact with the metal.

## 1 Introduction

The study of the role played by metal ions in biology dates back several decades. Among the metals normally present in living systems, transition metals, such as Fe, Co, Cu, and Zn, are the most noteworthy. They usually exist as electropositive chemical elements that can replace the proton of an acid and form complexes with hydroxyl anions. Metals are highly reactive and are involved in many important biological processes (Shi and Chance, 2008; Bertini et al., 2007; Crichton, 2019). An unbalanced concentration of metals can result in severe threats to living systems.

Many mechanisms make metal ions easily available when needed but harmless when stored. In many instances, whether metals are useful or harmful crucially depends on their local concentration. Thus, storing, metabolism, and trafficking of metals must be accurately regulated. The failure of the tuning mechanism of metal delivery is possibly also involved in the development of severe neurodegenerative pathologies such as Alzheimer’s disease, Transmissible Spongiform Encephalopathies, and Parkinson’s disease (De Santis et al., 2015).

One of the proteins required for the fine and correct tuning of cellular iron homeostasis is frataxin (FXN). FXN is highly conserved in eukaryotes and prokaryotes and plays a role in different cellular pathways. FXN specific function(s) is (are) still a matter of debate, while there is a general agreement that binding of iron to FXN is required for the cellular control of iron homeostasis. Nevertheless, the FXN role in helping cells regulate iron chemistry and availability has not been fully elucidated. Among many others, functions proposed for FXN are that it may act as an iron chaperone during heme and iron-sulfur (Fe-S) cluster assembly, serve as an iron-storage reservoir during iron overloading, and/or serve as a factor in lowering the concentration of reactive oxygen species (ROS), thus controlling the cellular oxidative stress (Bencze et al., 2006).

It is unlikely that only a single protein can control, in different cells, so many different pathways. In contrast, it is possible that different functions are not specific for FXN. Nevertheless, a breakdown in FXN production may lead to a complete failure in controlling the cellular iron availability. In particular, the deletion of the FXN gene has been seen to be followed by overloading of mitochondrial iron deposits and a general default of the Fe-S cluster assembly (Babcock et al., 1997; Foury and Cazzalini, 1997; Foury, 1999; Rötig et al., 1991). On these premises, FXN has been proposed as a candidate for the control of the mitochondrial iron efflux (Radisky et al., 1999).

Reduced expression levels of FXN in humans are linked to the development, progression, and severity of Friedreich’s ataxia (FA) (Pastore and Puccio, 2013), a neurodegenerative disease that is supposed to be linked to disproportion in iron concentration (Gentry et al., 2013).

The 210 amino acids long human FXN precursor is imported into the mitochondrion where it undergoes a two-step proteolytic maturation: first into a 19 kDa (42–210) intermediate and then into the final 14 kDa (81–210) form^1^, in both healthy individuals and FA patients (Pastore and Puccio, 2013).

FXN is known to possess non-structured regions (of which there is no 3D information from X-ray crystallography) that are supposed to play quite an important (direct/indirect) role in iron binding.

To date, FXN is not considered a cancer driver gene (Davoli et al., 2013; Martínez-Jiménez et al., 2020). However, it has been reported by Pastore and Puccio (2013) that, in cancer tissues, several FXN variants, whose stability and functional activity are reduced with respect to the wild-type (wt), are present. The importance of mutations in the onset and progression of neoplastic diseases is not yet clarified. However, mitochondrial functions and the complex process of tumorigenesis are connected at multiple levels. Impaired FXN functions may lead to defective mitochondria, which leads to a reduced assembly of Fe-S clusters, thus enhancing carcinogenesis (Rychtarcikova et al., 2017).

The Fe-FXN coordination mode is not fully elucidated. However, of the three putative ion binding sites, one is found to bring into play the His_86_ residue located in the disordered N-terminal region, while the other two involve aspartate and glutamate residues in a region of the protein called the “acidic ridge” (Gentry et al., 2013).

In this complex biological framework, the main goal of our research is to help understand the key structural aspects of the interaction of FXN and the three somatic missense variants of D104G, Y123S, and S161I (described in Section 2.2) with Fe^2+^ mimicked by its akin Co^2+^ (Maiti et al., 2017; Söderberg et al., 2011; Söderberg et al., 2013).

Following the same strategy already successfully used in by Gentry et al. (2013), Noguera et al. (2015), and Pastore et al. (2007), in the present study, we have replaced Fe^2+^ with the chemically very similar Co^2+^ ion. In fact, although the FXN natural ligand ion is Fe^2+^, the latter is known to be very unstable, spontaneously switching to the oxidized Fe^3+^ species in aerobic conditions. Instead, Co^2+^ is not sensitive to aerobic conditions and has been proved to be a reliable surrogate to probe Fe^2+^ sites in proteins (Maiti et al., 2017) and particularly in frataxin (Söderberg et al., 2011; Söderberg et al., 2013). The use of Co^2+^ in place of Fe^2+^ to study the structural properties is also supported by the observation that Co^2+^ has an ionic radius very similar to that of Fe^2+^ (Shannon, 1976).

The experimental technique of election for the study of protein-metal interactions is X-ray absorption spectroscopy (XAS) because, even in the case of very diluted metal-protein complexes, it allows providing accurate structural information about the environment of the metal-binding site within a radius of up to about 5 Å  (Strange et al., 1993; Ortega et al., 2012) ^2^.

In particular, XAS has shown its valuable power in elucidating the mechanisms by which metal ions interacting with intrinsically disordered proteins can push the latter to either the correct folding or a potentially dangerous misfolding, according to specific physicochemical circumstances (Morante et al., 2004; Morante and Rossi, 2014).

This work compares structural XAS information with data obtained from other complementary experimental techniques: circular dichroism, fluorescence spectroscopy and melting temperature measurements. A set of fairly good consistent results for the structure of the protein-metal complex and the stability ranking among wt-FXN and the three variants of D104G, Y123S, and S161I upon metal binding is obtained in this way.

## 2 Materials and Methods

### 2.1 Circular Dichroism and Fluorescence Spectroscopy

Circular dichroism (CD) and fluorescence spectroscopy (FS) techniques (Ősz et al., 2002) are used to monitor conformational changes induced by Co^2+^ binding in wt-FXN and in its variants D104G, Y123S, and S161I.

CD measurements were carried out with a JASCO-815 spectropolarimeter (Jasco, Easton, MD, United States), and the results are expressed as the mean residue ellipticity (Θ), assuming a mean residue molecular mass of 110 per amino acid residue.

Far-UV (190–250 nm) CD spectra were monitored at 20°C at an FXN concentration ranging from 100 to 140 μg/ml, using a 0.1 cm path length quartz cuvette. Near-UV (350–420 nm) CD spectra were recorded in a 1.0 cm path length quartz cuvette at a protein concentration ranging between 1.00 and 1.30 mg/ml. In both the far- and near-UV CD spectra measurements, the protein was dissolved in 20 mM Hepes, pH 8.0, 20 mM Na_2_SO_4_, and 20% glycerol.

Wild-type FXN and variants (with protein concentrations ranging from 100 to 140 μg/ml) were heated from 20 to 95°C and then cooled from 95 to 20°C in a 0.1 cm quartz cuvette with a heating rate of 1.0°/min controlled by a Jasco programmable Peltier element. The dichroic activity at 222 nm and the PMTV were continuously monitored in parallel every 0.5°C (Benjwal et al., 2006). All thermal scans were corrected for the solvent contribution at different temperatures. Melting temperature (Tm) values were calculated by taking the first derivative of the ellipticity at 222 nm with respect to temperature. All denaturation experiments were performed in triplicate.

Intrinsic fluorescence emission spectra were monitored at 50 μg/ml under the identical excitation and emission conditions for all the samples. Right angle light scattering was measured at 20°C with both excitation and emission wavelengths set at 480 nm. Measurements were performed using an LS50B spectrofluorometer (PerkinElmer) with a 1.0 cm path length quartz cuvette. Spectra were recorded in a range from 300 to 450 nm, with a 1 nm sampling interval, and the excitation wavelength was set at 295 nm.

### 2.2 X-Ray Absorption Spectroscopy

XAS measurements at the Co K-edge were performed at the BM30 beamline of the European Synchrotron Radiation Facility (ESRF; Grenoble, France) Proux et al. (2005).

The beam energy was selected using a Si(220) double-crystal monochromator with a resolution of 0.5 eV. The beam spot on the sample was approximately 240, ×, 150 *μ*m^2^ (H × V, FWHM). Spectra were recorded in fluorescence mode using a 13-element solid-state Ge detector. To avoid photo-degradation and undesired spectra evolution during data taking, all the samples, held at 77 K since their preparation, were cooled down and kept at 13 K in a helium cryostat during the whole XAS measurements. For the same reason, in the process of data acquisition, the hitting X-ray beam was systematically moved to a different position on the sample after each scan.

As usual, XAS spectra are analyzed by separating the low photons energy XANES (X-ray absorption near edge spectroscopy) region from the high photons energy EXAFS (extended X-ray absorption fine structure) one. The XANES spectra are normalized using the standard software Athena (Ravel and Newville, 2005). EXAFS data are extracted with the help of cubic splines interpolation as implemented in the AUTOBKG algorithm (Newville et al., 1993) of Athena. EXAFS data analysis was performed using the EXCURV98 code (Binsted et al., 1998).

**FIGURE 1:**
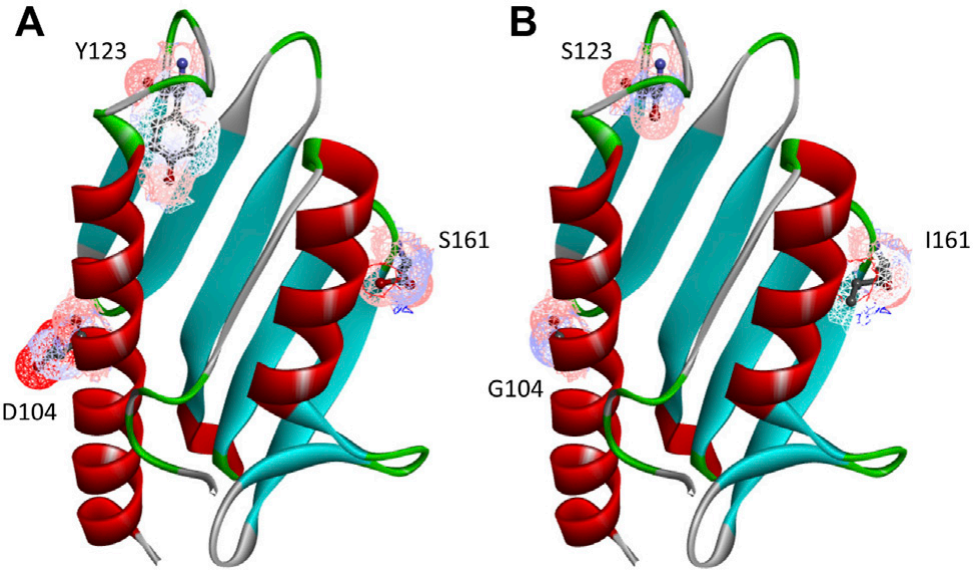
Human FXN folded structure. The three point-mutations, D104G, Y123S, and S161I, which are the object of this investigation, are highlighted. **(A)** Wild-type, **(B)** variants.

As specified in Table 1, besides the wt-FXN in two different lengths, we considered three somatic missense variants (Petrosino et al., 2019): D104G, Y123S, and S161I. These mutations, whose location in the folded protein is highlighted in Figure 1, have different effects on the Fe-S cluster formation, as shown by absorbance measurements by Petrosino et al. (2019). The reasons for focusing on these somatic missense variants are summarized as follows:

• D104G: this variant is present in liver carcinoma cells. As shown in Figure 1, residue D104 is located in the *α*_1_ helix region^3^ of the protein. In the case of this variant, Fe-S clusters formation is totally inhibited, although the thermodynamic stability and the secondary and tertiary structure arrangements are similar to those of the wt-FXN (Petrosino et al., 2019).
• Y123S: this variant is present in the cells of the digestive tract carcinoma. Residue Y123 is located in the coil region between the *β*-sheet region and the *α*_1_ helix (Figure 1). In this case, activity measurements show that Fe-S clusters formation is only partially inhibited. This FXN variant shows a Tm of about 14°C lower than that of the wt-FXN, suggesting an enhanced unfolding propensity and a significant decrease in thermal stability (Petrosino et al., 2019).
• S161I: this variant has been found in the cells of the endometrium carcinoma. Residue S161 is located in the coil region between the *β*-sheet region and the *α*_2_ helix. Again the thermal stability is decreased, as indicated by a Tm lower, by about 11°C, compared to that of the wt-FXN. Interestingly, with this missense mutation, the Fe-S cluster formation is totally inhibited (Petrosino et al., 2019).

**TABLE 1:** In the first column, we report the names by which the analyzed samples are denoted in the study.

Name	81–210	90–210	(Co) eq
wt_90_none		*	None
wt_90_0.8		*	0.8
wt_90_1.6		*	1.6
wt_81_none	*		None
wt_81_0.3	*		0.3
wt_81_0.8	*		0.8
D104G_none	*		None
D104G_08	*		0.8
Y123S_none	*		None
Y123S_08	*		0.8
S161I_none	*		None
S161I_08	*		0.8

Asterisks in the following two columns identify the portion of the amino acid sequence of the FXN protein as either 81-210 (first column) or 90-210 (second column). In the last column, the Co^2+^ concentration in protein equivalent (0.3, 0.8, and 1.6) is reported, while “none” stays for the absence of metal. Of course, this last set of samples, lacking the metal absorber, was not submitted to any XAS measurement.

For the purpose of XAS measurements, wt-FXN and variants have been expressed as N-terminal His-tagged proteins using a pET28a vector in *E. coli* Rosetta cells transformed with the selected plasmid, grown in LB medium, and purified as described by Petrosino et al. (2019).

The samples listed in Table 1 were prepared for the XAS measurements according to the following protocol. For the wt protein and its variants, stocks of 45 μL of 0.9 mM protein solutions were obtained by dissolving the appropriate amount of protein in 20 mM Hepes, pH 8.0, containing 20 mM Na_2_SO_4_ and 20% glycerol. A Co^2+^ 16 mM stock solution was prepared by dissolving CoSO_4_ in a 20 mM Hepes, pH 8.0, containing 20 mM Na_2_SO_4_ with 20% glycerol. In order to get the desired Co^2+^ final concentrations (see Table 1), to each 45 μL sample holder, the appropriate amount of a Co^2+^ concentrated stock solution was finally added. The solutions in each sample holder were mixed by pipetting and then immediately frozen in liquid N_2_, shipped overnight from Rome to Grenoble in a dry shipping Dewar cooled with liquid N_2,_ and immediately transferred to the ESRF beamline upon arrival.

## 3 Results

### 3.1 Circular Dichroism, Fluorescence Spectroscopy, and Thermal Shift Analysis

Both near-UV CD and FS suggest that Co^2+^ interacts preferentially with the short FXN isoform 90-210 rather than with the long isoform 81-210.

**FIGURE 2:**
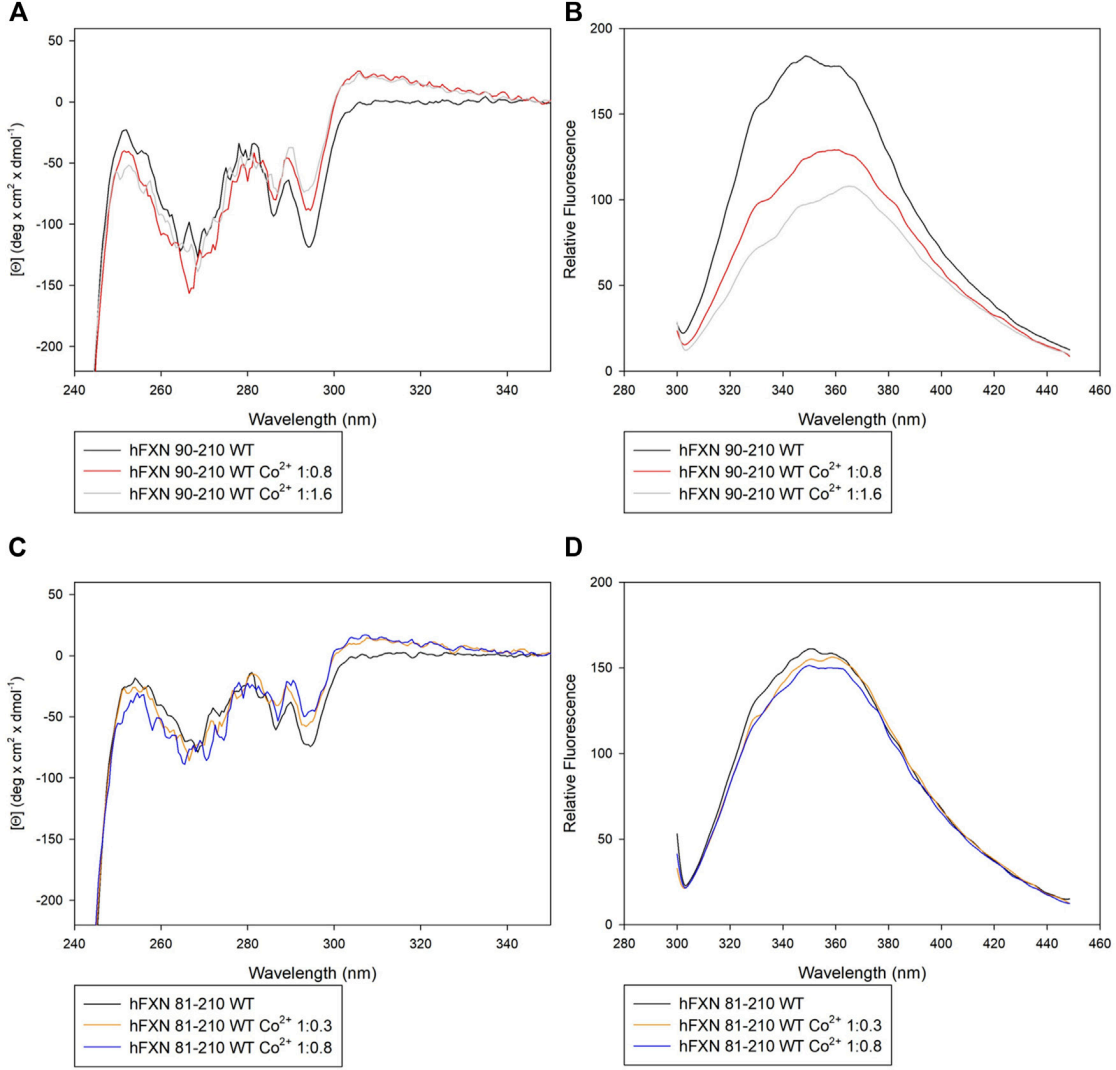
Spectroscopic characterization of FXN isoforms with and without Co^2+^. **(A)** Near-UV CD spectra of FXN 90-210 in the absence of Co^2+^ (black) and in the presence of 0.8 (red line) and 1.6 (grey line) Co^2+^ equiv. **(B)** Intrinsic fluorescence emission spectra of FXN 90-210 in the absence of Co^2+^ and in the presence of 0.8 and 1.6 Co^2+^ equiv (code color is as in panel **A**). **(C)** Near-UV CD spectra of FXN 81-210 in the absence of Co^2+^ (dark purple) and in the presence of 0.3 (orange) and 0.8 (blue) Co^2+^ equiv. **(D)** Intrinsic fluorescence emission spectra of FXN 81-210 in the absence of Co^2+^ and in the presence of 0.3 and 0.8 Co^2+^ equiv (code color is as in panel **C**). Fluorescence spectra were monitored at 50 μg/ml under identical excitation and emission conditions.

In particular, panel **A** of Figure 2 shows that, by adding Co^2+^ to FXN 90-210, important changes occur in the regions of 252–256, 265–270, and 288–295 nm. At the same time, fluorescence intensity reduction and redshift are observed (see panel **B** of Figure 2), indicating consistent conformational changes that may be related to the Co^2+^ presence with protein-metal complex formation. Right angle light scattering data indicate that aggregation never occurred in any measured samples.

Furthermore, the longer, FXN 81-210, isoform shows changes in the near-UV CD spectrum in the regions 252–256 and 288–295 nm (see panel **C** of Figure 2). However, neither fluorescence intensity modification nor redshift is visible (panel **D**), thus hinting at a lack of protein-metal complex formation.

**FIGURE 3:**
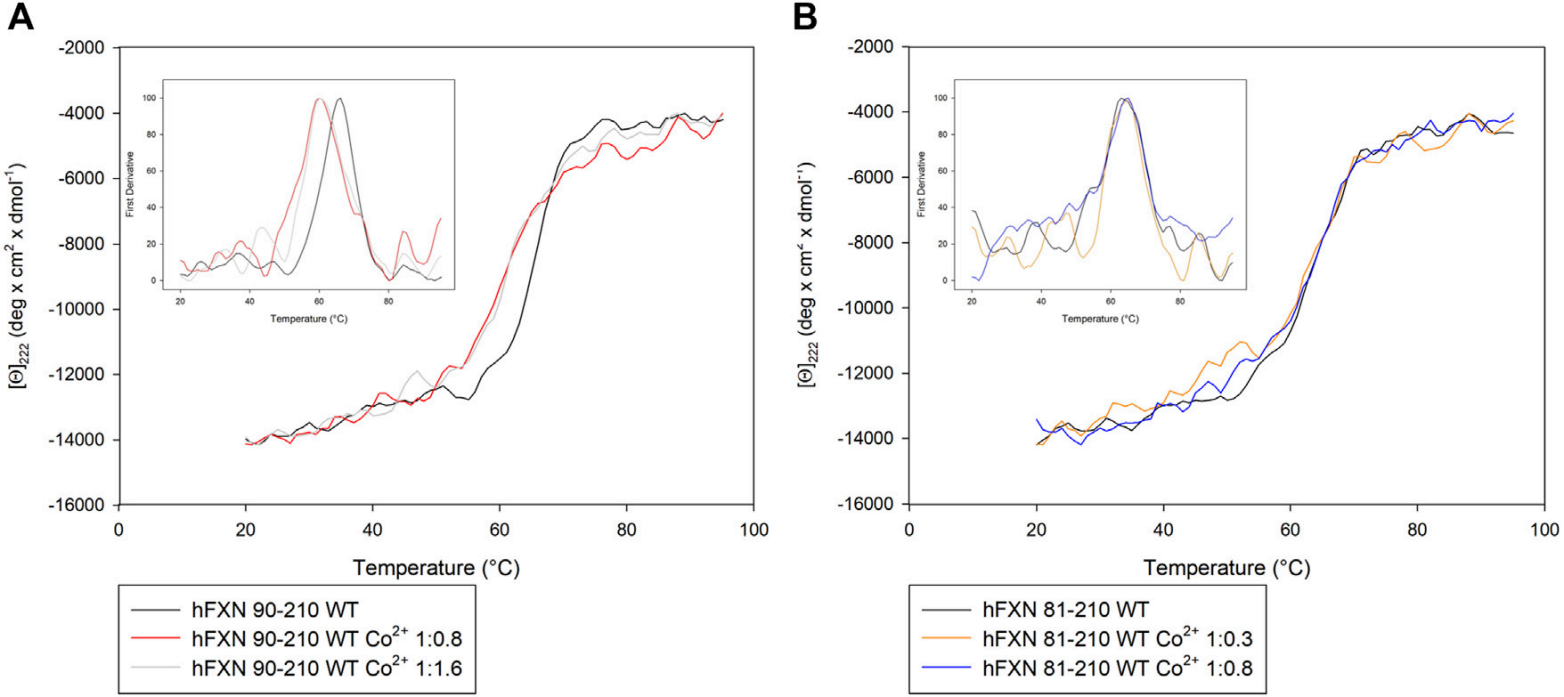
Thermal unfolding transition of FXN isoforms with and without Co^2+^. **(A)** Thermal unfolding curves of FXN 90-210 in the absence of Co^2+^ (black) and in the presence of 0.8 (red) or 1.6 (grey) Co^2+^ equiv. **(B)** Thermal unfolding curves of FXN 81-210 in the absence of Co^2+^ (dark purple) and in the presence of 0.3 (orange) or 0.8 (blue) Co^2+^ equiv. The curves are obtained by monitoring the molar ellipticity at 222 nm [Θ]_222_, in the range between 20 and 95°C. Insets show the first derivative of the experimental curve.

These indications are confirmed by thermal stability analysis performed on the protein in the presence of Co^2+^ at the concentrations specified in Table 2 (see Figure 3). Thermal stability measurements are considered a powerful and reliable way to study metal-binding interactions in proteins (Layton and Hellinga, 2011).

**TABLE 2:** Melting temperature of wt-FXN and D104G, Y123S, and S161I variants, in the absence of Co^2+^ and in the presence of 0.8 and 1.6 Co^2+^ equiv. Sample names are as shown in Table 1.

Sample name	Tm (°C)
wt_81_none	65
wt_81_0.3	65
wt_81_0.8	65
wt_90_none	66
wt_90_0.8	60
wt_90_1.6	60
D104G_90_none	68
D104G_90_0.8	63
Y123S_90_none	53
Y123S_90_0.8	Tm_1_ = 43; Tm_2_ = 59
S161I_90_none	56
S161I_90_0.8	Tm_1_ = 46; Tm_2_ = 54

In order to measure thermal stability, samples with the wt-FXN and samples with the D104G, Y123S, and S161I variants at concentrations ranging from 100 to 140 μg/ml, in the absence of Co^2+^ and the presence of different concentrations of Co^2+^, were heated from 20°C to 95°C in a quartz cuvette with a heating rate of 1°C/min, controlled by a Jasco programmable Peltier element as described by Petrosino et al. (2019). We stress that thermal transitions appeared reversible in all the conditions we considered, with no hysteresis during the cooling phase (see Supplementary Material).

**FIGURE 4:**
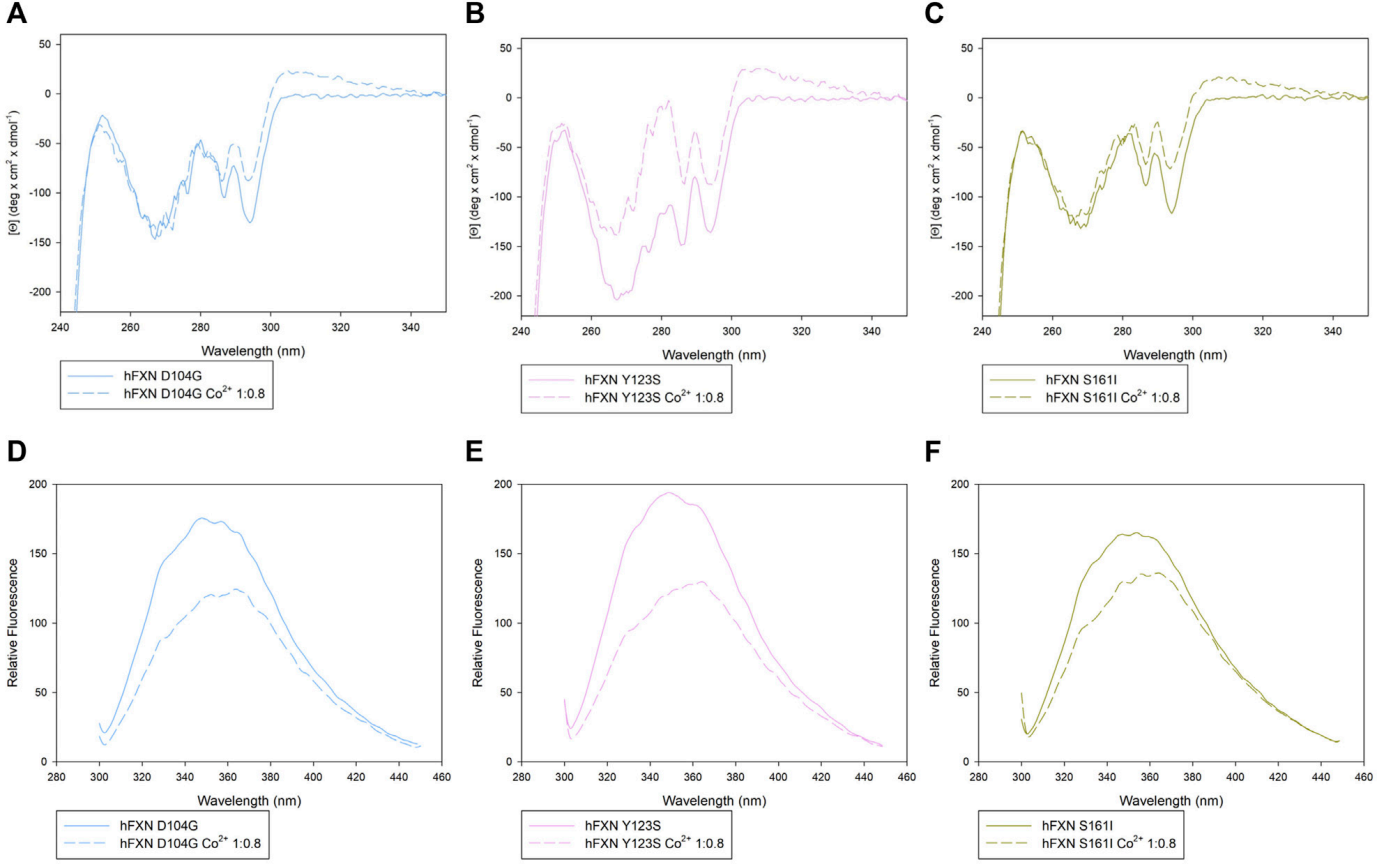
Spectroscopic characterization of the D104G, Y123S, and S161I variants with and without Co^2+^. Upper panels: near-UV CD spectra of D104G (light blue, **A**), Y123S (light pink, **B**), and S161I (dark yellow, **C**) in the absence of Co^2+^ (continuous line) and in the presence of 0.8 Co^2+^ equiv (dotted line). Lower panels: intrinsic fluorescence emission spectra of D104G (light blue, **D**), Y123S (light pink, **E**), and S161I (dark yellow, **F**) in the absence of Co^2+^ (continuous line) and in the presence of 0.8 Co^2+^ equiv (dotted line). Fluorescence spectra were monitored at 50 μg/ml under identical excitation and emission conditions.

**FIGURE 5:**
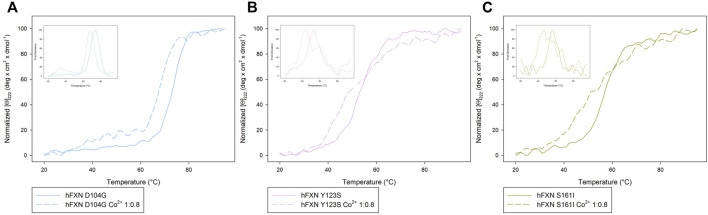
Comparison of the Near-UV CD data of [Θ]_222_ as a function of the temperature of the D104G (light blue), Y123S (light pink), and S161I (dark yellow) variants **(A–C)** in the absence of Co^2+^ (continuous curves) and in the presence of 0.8 Co^2+^ equiv (dotted lines). The insets show the first derivative of the experimental curve with respect to temperature.

Looking at the behavior of the molar ellipticity changes at 222 nm between 20°C and 95°C, we observe a decrease in the Tm values of the wt sample by 6° upon adding 0.8 equiv or 1.6 Co^2+^ equiv to FXN 90-210 (Table 2; Figure 3A). Instead, Tm does not change when the metal ion is added to FXN 81-210 (see Table 2; Figure 3B). The three variants, D104G, Y123S, and S161I, show conformational changes and a decrease in Tm in the presence of Co^2+^ (see Figures 4, 5).

In the case of the Y123S and S161I variants, two melting temperatures are reported in Table 2. They correspond to the two local maxima displayed by the dotted curves in the insets of Figures 5B,C. The presence of two slopes in the Θ_222_ curve is interpreted as an indication of the existence of a two-step thermal denaturation process.

The most significant structural modification induced by Co^2+^ was observed in the case of the Y123S variant, which involves the tyrosine residue in position 123, in a region usually considered a putative FXN iron-binding site (Nair et al., 2004; Gentry et al., 2013).

In the case of the Y123S variant, the presence of Co^2+^ induces a dramatic modification in both the near-UV CD and the fluorescence spectra (Figures 4B,E). Indeed, in the presence of 0.8 Co^2+^ equiv, a significant loss of cooperativity occurs in the thermal unfolding process, as shown by the far-UV CD data of [Θ]_222_ as a function of the temperature reported in Figures 5B,C and Table 2.

### 3.2 X-Ray Absorption Spectroscopy

Figure 1 displays the structured region of wt-FXN and the short non-structured region at the C-terminal of the amino acid sequence. A second disordered region is located between residues 81 and 90^4^. For the latter, we propose a peculiar role in modulating metal binding (see Section 4) based on the analysis of XAS data (see below) and the melting temperature results reported in Table 2.

The first step toward a correct interpretation of the physicochemical implications of the FXN-metal interaction is to clarify the structure of the accessible metal-binding sites located along the protein amino acid sequence. For this task, XAS is the technique of election as it allows accurately “taking a picture” of the metal ion atomic environment within a region of up to about 5 Å around the metal absorber.

As customarily done, we shall separately discuss the features of the low energy (XANES) and the high energy (EXAFS) region of the XAS spectrum.

The XANES region is dominated by multiple scattering processes, where the photoelectron undergoes more than one scattering event against the atomic scatterers located within a sphere of a radius of about 5 Å from the absorber. Although extracting accurate geometrical information from the XANES region of the spectrum is computationally extremely difficult, fingerprints of specific geometries can still be identified (see Section 3.2.1). In the EXAFS region, where the extracted photoelectron owns a quite high kinetic energy, single scattering events prevail, thus making, in principle, the extraction of geometrical information comparatively much easier (see Section 3.2.2).

#### 3.2.1 XANES

In this section, we discuss the information one can get from comparing the XANES spectrum of the wt-FXN with the three variants we are focusing our attention on.

**FIGURE 6:**
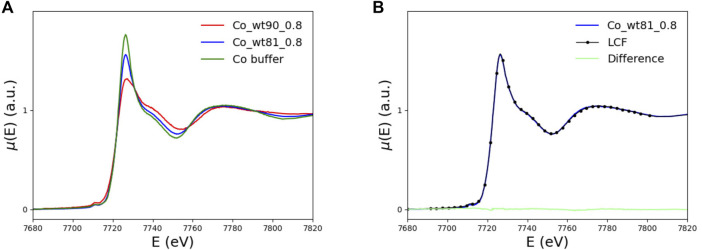
**(A)** XANES spectra of the FXN samples including (wt_81_0.8, blue curve) and not including (wt_90_0.8, red curve) the N-terminal 81-90 disordered region, compared with the buffer spectrum (green curve). **(B)** XANES spectrum of the long, wt_81_0.8, fragment (blue curve) compared to the spectrum obtained as a linear combination of 43% of wt_90_0.8 and 57% of buffer spectra (black dotted). In light green, we plot the difference spectrum.

In the left panel of Figure 6, the XANES spectra of the wt_81_0.8 and wt_90_0.8 samples are compared with the buffer spectrum. The two peptides differ because of the presence (wt_90_0.8) or absence (wt_81_0.8) of the disordered 81-90 tail.

We recall that the His_86_ amino acid, which is supposed to be a binding site for Co^2+^ (as well as Fe^2+^) (Gentry et al., 2013), is missing in the shorter fragment wt_90_0.8.

By comparing, in the left panel of Figure 6, the XANES data for the wt_81_0.8 sample with those of the wt_90_0.8 sample and the buffer, one sees that the spectrum of the longer fragment, wt_81_0.8 sample, looks much more similar to the buffer than the spectrum of the wt_90_0.8 sample. This feature suggests that, in the case of wt_81_0.8, a non-negligible amount of Co^2+^ remains free in the solution.

In order to make this observation more quantitative, we perform a minimization of the difference between the wt_81_0.8 XANES spectrum and the spectrum obtained as a linear combination, point by point, of the wt_90_0.8 and buffer spectra. By fitting the mixing coefficient, we find that the wt_81_0.8 XANES spectrum is well reproduced by a linear combination of 43% of the wt_90_0.8 spectrum plus 57% of the buffer.

We conclude that, in the case of the wt_81_0.8 sample, more than half (57%) of the available Co^2+^ does not bind to FXN but remains free in solution. This observation is the reason why we did not explore higher metal concentrations. In fact, going at 1.6 eq (or higher) would only increase the uninteresting contribution of Co^2+^ in the solution.

This finding seems somewhat surprising because it says that, in the presence of an extra putative metal-binding site, a smaller amount of Co^2+^ is bound to the protein.

We will show in Section 4 that the reason for this, at first sight, strange feature has to do with the peculiar arrangement of the disordered 81-90 tail belonging to the longer wt_81_0.8 fragment. The point is that this tail can “obscure” some of the protein metal-binding sites otherwise available for binding in the case of the shorter wt_90_0.8 peptide (see Figure 13). This interesting interpretation of the puzzling XANES data in Figure 6 is nicely confirmed by the results of melting temperature measurements displayed in Table 2, which show that, in the presence of the metal, the longer 81-210 fragment is more stable than the shorter one.

**FIGURE 7:**
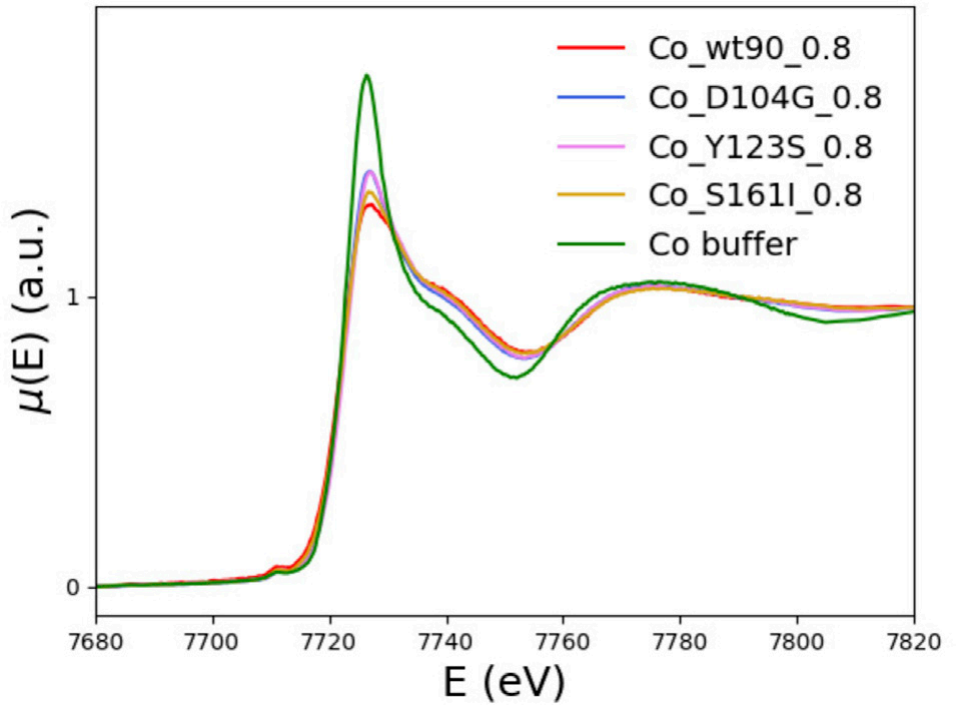
The XANES spectra of the D104G (blue), Y123S (violet), and S161I (gold) variants are plotted together with the wt-FXN (red) and the buffer (green) spectra.

In Figure 7, the XANES spectra of the D104G (blue), Y123S (pink), and S161I (gold) variants are compared with those of the wt_90_0.8 sample (red) and the buffer (green). We see that the buffer spectrum appears to be significantly different from the spectra of all the variants, thus concluding that all the variants can bind Co^2+^.

The tendency of XANES spectra of D104G and Y123S variants toward that of the buffer indicates a slightly larger contribution to spectra of dissociated forms in these variants than in the wild-type sequence.

Qualitatively, we see that the most significant difference with the wt-FXN spectrum occurs in the case of variants, in which the destabilizing effect due to metal binding (measured by the difference of the melting temperature with and without metal, see Table 2) is large. While destabilization upon metal binding is somewhat surprising in the case of the D104G mutation, as the latter just occurs well inside the alleged metal-binding acidic ridge, the other two mutations, Y123S and S161I, are located far away from it, and the reasons at the basis of the metal-induced destabilization are more difficult to identify. They would require a much more refined investigation, which is outside the scope of the present work.

#### 3.2.2 EXAFS

**FIGURE 8:**
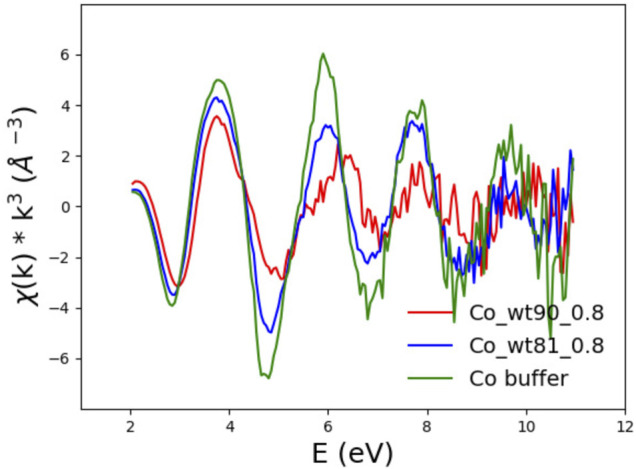
EXAFS spectra of FXN wt_90_0.8 (red) and wt_81_0.8 (blue) in the presence of 0.8 Co^2+^ equiv compared to the buffer spectrum (green).

The first simple observation is that EXAFS spectra show the same ranking of similarity observed for the XANES spectra. In particular, one notices that although the EXAFS spectra of the wt_90_210 and wt_81_210 samples are both definitely different from that of the buffer (see Figure 8), they are also quite different between themselves, with the spectrum of the wt_81_210 sample visibly more similar to that of the buffer than to that of the wt_90_210 sample.

Structural information about the atomic arrangement around the absorbing metal can be extracted from a fit to the EXAFS region of the spectrum, provided that some “reasonable” assumption about the atomic structure around the Co^2+^ absorber is available. Unfortunately, not much is known about the precise location of the possible Fe/Co-FXN binding sites (Gentry et al., 2013). In order to overcome this lack of information, we decided to take advantage of the metal ion-binding (MIB) site prediction and docking server (Lin et al., 2016) that, given the protein 3D structure, provides the most probable binding sites for a specific metal ion.

The amino acid patterns to which MIB has assigned the highest scores for Co^2+^, Fe^2+^, and Fe^3+^ binding in FXN 90-210 are reported in Table 3. As expected, the so-called acid ridge is signaled as the most probable region for Co^2+^, Fe^2+^, and Fe^3+^ binding.

**TABLE 3:** Pairs of residues to which MIB assigns the highest score as Fe^2+^, Fe^3+^, and Co^2+^ metal-binding sites in wt-FXN 90-210.

FXN 90-210		
Fe^2+^	Fe^3+^	Co^2+^
Asp_104_-Glu_108_	Glu_108_-Glu_111_	Glu_100_-Asp_104_
Glu_96_-Glu_100_	Asp_104_-Glu_108_	Glu_108_-Asp_112_

We limited the MIB search to FXN 90-210 because the FXN 81-210 longer fragment differs from the former for the presence of the disordered 81-90 N-terminal tail, which is not supposed to be able to host a metal. Despite this fact, as discussed in Section 4, this unstructured protein region will be argued to play an important role in the FXN 81-210 ability to bind the metal.

Building on the structural information provided by the MIB search, we are in a position to construct a physically sensible Co^2+^ binding model configuration from which one can start the theoretical calculation of the EXAFS spectrum. Fitting model parameters against the measured spectrum will allow for accurately determining the atomic structure of the metal-binding site.

As a starting configuration, we took the one with the highest score among those listed in the last column of Table 3. It corresponds to a situation in which Co^2+^ is coordinated to the residues Glu_100_ and Asp_104_.

**FIGURE 9:**
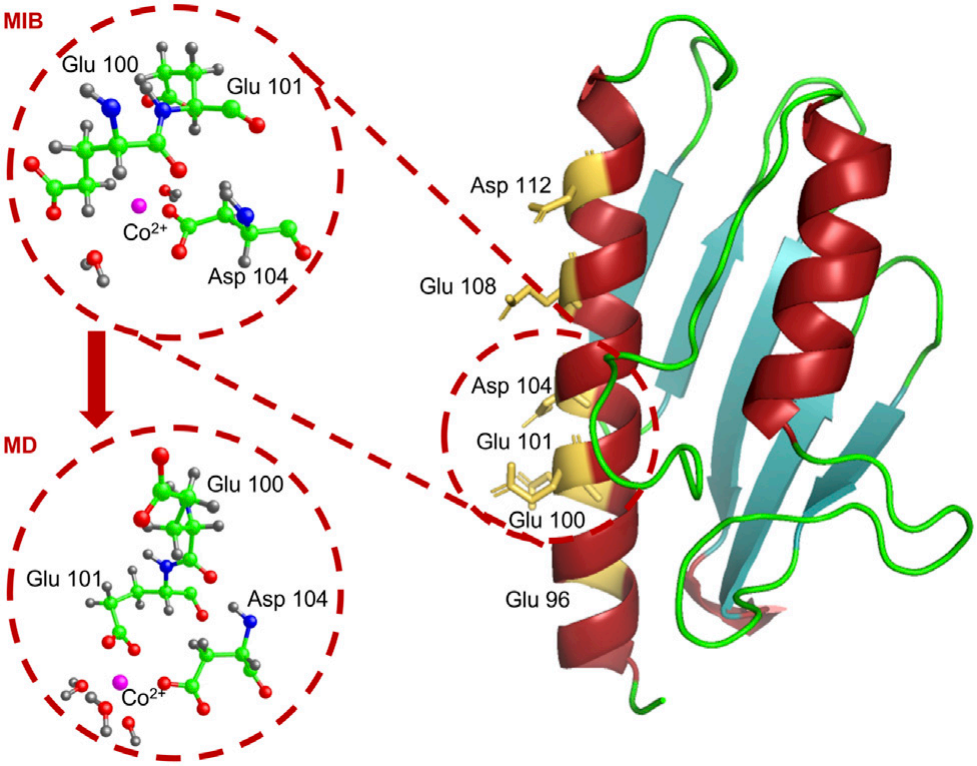
We show the atomic structure of the Glu_100_-Asp_104_ site around the Co^2+^ ion, as proposed by MIB (upper circle) and at the end of the MD simulation (lower circle). Co^2+^ is in pink, carbon in green, oxygen in red, nitrogen in blue, and hydrogen in grey.

The structure provided by MIB (see the upper circled inset in the left part of Figure 9) is relaxed and equilibrated at 300 K with classical MD simulations.

MD simulations were performed using the code NAMD (Phillips et al., 2020) with the Co^2+^ ion forced in its preferred octahedral geometry by employing the *dummy atoms* method (Pang, 2001; Furlan et al., 2010; La Penna et al., 2013; Duarte et al., 2014; La Penna et al., 2015). Indeed, the octahedral Co^2+^ geometry, which is at the basis of our MD simulations, is confirmed by the pre-edge feature visible in the XANES spectra of Figure 7, around 7,710 eV (signaling a 1 s → 3 d transition), typical of an octahedral (or pseudo octahedral) site geometry (Bresson et al., 2006).

The *dummy atoms* method amounts to perform MD simulations where the positive density of the divalent cation is distributed in a blocked octahedral geometry to prevent the negatively charged groups from collapsing over the charged point-like particle of the opposite sign (La Penna and Chelli, 2018).

The metal-protein complex was placed in a 64 × 62 × 74 Å^3^ box and solvated by adding 9259 TIP3P water molecules to have a layer of water of at least 15 Å around the protein. Na^+^ and Cl^−^ ions were inserted in the box to get an overall neutral system with the same ion concentration as in the *in vitro* experiments. At the end of a 4 ns MD simulation, Co^2+^ is found to be stably located at a binding distance from the Glu_101_ and Asp_104_ residues (see lower circled inset in the left panel of Figure 9), thus slightly displaced from its initial position.

A sphere of a 5 Å radius, centered on the Co^2+^ ion, is excised from the simulation box and used as the starting configuration for the fit of the EXAFS data of the *wt*_90_08 sample.

The fit to the EXAFS data is performed by employing the program EXCURV98 Binsted et al. (1998). The code implements the so-called constrained refinement strategy, which consists of treating molecules as rigid bodies. In this way, the number of fitting parameters can be significantly reduced (Binsted et al., 1992). In the present case, we considered the amino acids bound to the Co^2+^ ion rigid bodies, namely, Glu_101_ and Asp_104_. Starting from the configuration depicted in the lower circled inset in Figure 9, we refined the Fermi energy shift and the distances of the six oxygen atoms located in the first coordination sphere. These are the two oxygen atoms belonging to the carboxylic group of Glu_101_, the oxygen belonging to Asp_104_, and three oxygen atoms belonging to the three nearby water molecules (see Table 4).

**TABLE 4:** Co^2+^-ligand distances and Fermi energy shift at the end of the EXAFS fit. Note that the carboxylic group of Glu_101_ contributes to the octahedral coordination with two oxygen atoms.

Coordinated atoms	*r* ± Δ *r* (Å)
4 O (Glu_101_ + Asp_104_ + 2 water molecules)	2.02 ± 0.02
1 O (Glu_101_)	2.21 ± 0.01
1 O (water)	1.85 ± 0.02
E_*F*_ = (3 ± 1) eV	R-factor = 35%

**FIGURE 10:**
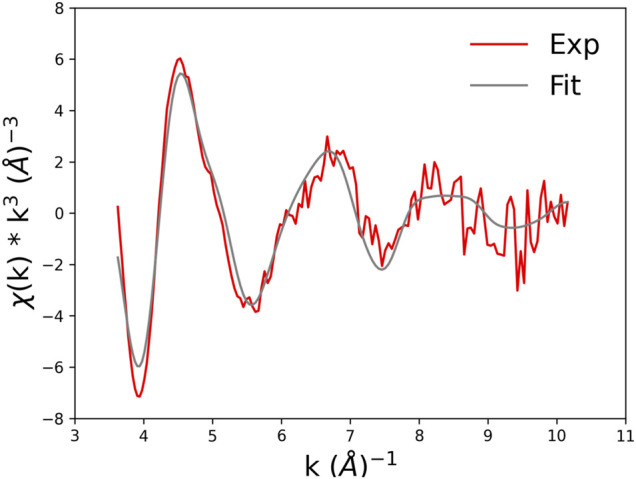
Experimental EXAFS spectrum of wt_90_0.8 (red curve) and best-fit model (grey curve).

**FIGURE 11:**
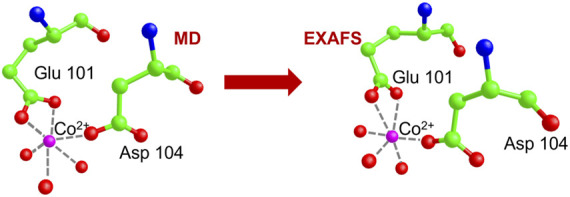
Comparison of the Co. binding site configuration at the end of the MD simulation and after the EXAFS fitting step. The color code is as shown in Figure 9. Hydrogen atoms are not shown as they are standardly considered not to contribute to the XAS signal. Broken lines represent the distances of Co^2+^ from its six oxygen ligands.

The best fit is displayed in Figure 10, where we show the experimental EXAFS data (in red) superimposed on the fitted curve (in grey). Table 4 gives the O-Co^2+^ best fit distances. Figure 11 compares the metal-binding site configuration at the end of the MD simulation and after the EXAFS fitting step. We remark that the MD simulation was able to give a pretty good picture of the atomic environment around the metal, as one finds that both before and after the EXAFS fit the six metal ligands (two oxygen atoms from Glu_101_, one atom from Asp_104_, and three atoms from water molecules) all fall in a shell between 1.8 and 2.2 Å.

We end this section with an observation concerning the second (putative) metal-binding site (Glu_108_-Asp_112_) identified by the MIB prediction and docking server (see Table 3). We note that an analysis completely analog to the one performed in the case of the Glu_100_-Asp_104_ site shows that the atomic environments of the two sites around the metal are practically identical (data not shown). The main reason is that, in the two sites, the same two ligands, namely, Glu and Asp, *via* the carboxylate group of the acid chain, are involved in metal binding. Thus, we expect that although the two ligands at the end of the MD simulation can be differently positioned in space, the two binding sites will equally contribute to the EXAFS experimental signal.

## 4 The Role of the Disordered 81-90 N-Terminal Region

It is very instructive to look at the XANES spectral regions (see Figure 6) in Section 3.2.1 in conjunction with the pattern of the measured melting temperature of wt-FXN and D104G, Y123S, and S161I variants (see Table 2), as quite an interesting feature emerges.

To start the discussion, we recall that the XANES spectrum of the 81-210 fragment is very well reproduced by the sum, point by point, of 43% of the wt_90_0.8 spectrum plus 57% of the buffer spectrum (see Section 3.2.1). This suggests that more than half of Co^2+^ remains in the solution; it is not bound to the protein.

**FIGURE 12:**
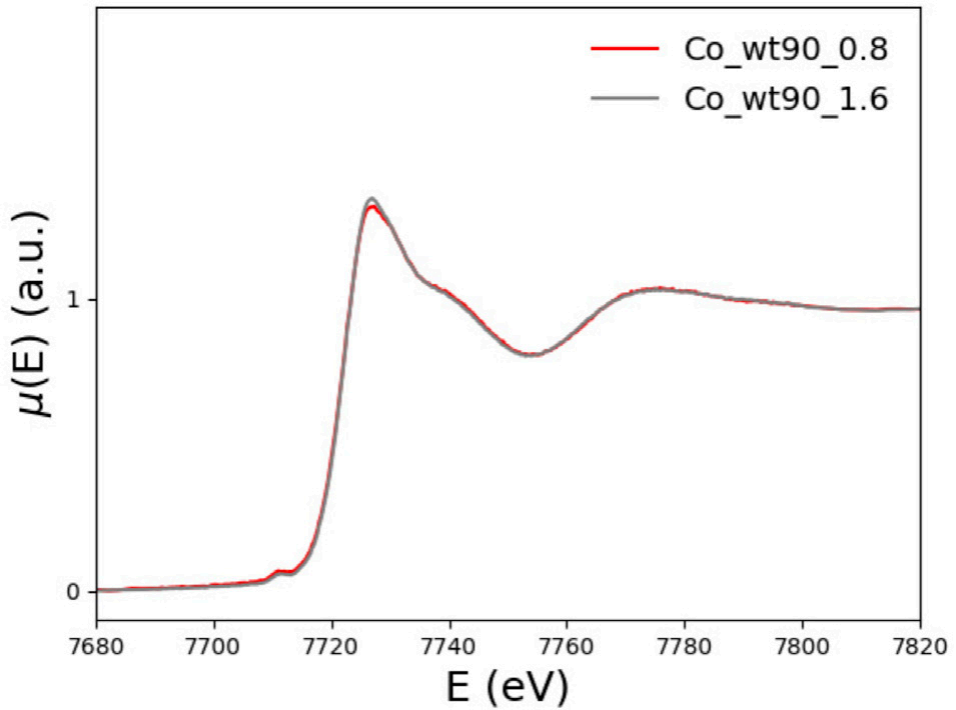
XANES spectra of wt_90_0.8 (red) and wt_90_1.6 (grey) in the presence of 0.8 and 1.6 Co^2+^ equiv, respectively.

We also note that, on the one hand, as Figure 12 shows, the XANES spectra of the 90-210 wt-FXN samples with 0.8 and 1.6 Co^2+^ equiv are well superimposable, proving that, even when the Co^2+^ is added to the solution at two times the protein concentration, still there is no Co^2+^ in the solution. This hints at the conclusion that at least two (more or less equivalent) Co^2+^ binding sites are available in the wt-FXN protein. On the other hand, it is necessary to assume that about half of the amount of Co^2+^ in the sample has remained free in solution (i.e., not bound to the protein) to get a good fitting of the XANES and EXAFS spectra of the 81-210 FXN.

A suggestive explanation for this rather puzzling behavior could be that the 81-90 tail present in 81-210 FXN somehow prevents Co^2+^ from binding to one of the two available coordination sites present in the acidic ridge.

### 4.1 Experimental Evidence

**FIGURE 13:**
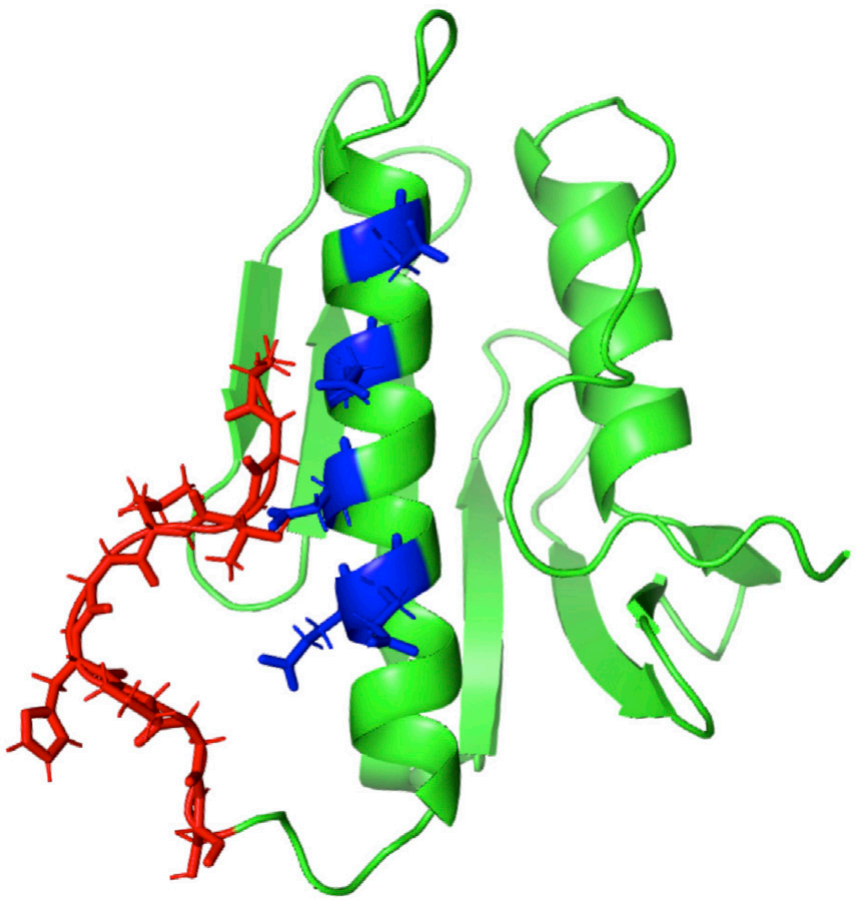
We display the configuration of the 81-90 disordered region in red as it results after an elaborated MD relaxation step that was started from an *all-trans* geometry. The potential metal-binding site locations are highlighted in blue.

The disordered 81-90 N-terminal tail in one of its relaxed configurations is geometrically long enough to be able to somehow “obscure” part of the acidic ridge in the *α*_1_ helix. In Figure 13, we show one of these typical configurations. They are obtained at the end of a long and careful MD simulation started from an initial *all-trans* arrangement.

This elongated configuration can result from the “sealing” of the Co^2+^ ion acting between the acidic ridge and the carboxylic end, which helps stabilize the structure. Such a model would explain why, in the case of the long 81-210 wt-FXN, half of Co^2+^ is found free in solution when the latter is added to the solution at a concentration of twice that of the protein.

The scenario we propose is confirmed by the pattern of the Tm values shown in Table 2. In fact, while the presence of Co^2+^ does not affect the Tm of the long, 81-210, wt-FXN fragment, it significantly destabilizes the short one, 90-210^5^. This observation reinforces our hypothesis, according to which, in the presence of the 81-90 disordered tail, metal ion binding sites in the acidic ridge are not fully available. As a result, the destabilizing effect due to metal binding is reduced or not occurring anymore.

## 5 Conclusion

Based on information extracted from some experimental techniques (CD, FS, XAS) and numerical simulations, we confirm the ability of the wt-FXN and its variants to stably bind metal ions (in our investigation Co^2+^), strengthening in this way the conjecture according to which FXN is involved in Fe^2+^ storage and transport.

In particular, XAS data consistently confirm the ranking of thermal stability established by measuring the melting temperatures when wt-FXN is compared to D104G, Y123S, and S161I variants.

Starting from the crystallographic structure of the putative Co^2+^ binding sites identified by the MIB site prediction and docking server (Lin et al., 2016), we have performed long (up to 4 ns) MD simulations of FXN in the presence of Co^2+^ to have a reliable atomic geometry around the metal that could be used as an initial configuration in the analysis of our newly collected XAS data. A fit to the EXAFS region of the spectrum allows us to positively identify the FXN acidic ridge as the location of the most likely metal-binding sites.

Furthermore, we can explain the surprising feature emerging from a detailed analysis of the XANES region of the spectrum, according to which the longer 81-210 FXN fragment has a smaller propensity for Co^2+^ binding than the shorter 90-210 one, despite the presence in the former of the His_86_ residue, which is supposed to be a specific Co^2+^ binding site (Gentry et al., 2013).

Indeed, the difference between the XAS spectrum of the long 81-210 FXN fragment compared to that of the shorter 90-210 one that we understand as due to a large fraction (almost a half) of Co^2+^ remaining in solution in the case of 81-210 FXN, is explained as due to a peculiar behavior of the “disordered” N-terminal region of 81-90 FXN. Our conjecture, which to our knowledge was never considered in the literature, is that the disordered N-terminal tail in the 81-210 FXN sample gets locked in an extended configuration by binding a Co^2+^ ion, which acts as a bridge between the 81-90 tail and the *α*_1_-helix. In this configuration, after a first metal ion is bound, the other metal-binding site allegedly present in the acid ridge is “obscured” by the extended N-terminal tail and becomes unattainable by a second metal ion. The Co^2+^ “sealing” ability we are invoking here is in agreement with the large body of experimental work that assigns a role to metal ions as structural stabilizers in disordered peptides and protein folding processes (see La Penna and Morante (2021) for a recent review on this issue).

In the future, we intend to develop our investigations along two lines. The first is a simulation study of the relative stability of wt-FXN compared to that of a more extended set of FXN single-point variants (Botticelli et al., 2022) than the ones considered here. The computation is carried out using the MD methods where altruistic metadynamics is employed to generate maximally unbiased sets of protein configurations (Barducci et al., 2006; Laio and Gervasio, 2008). The constrained maximal entropy principle is then exploited to appropriately reweigh the collected configurations in thermal average computations (La Penna et al., 2004).

The second line of investigation is of a more experimental nature and aims to extend to the FXN variants considered by Botticelli et al. (2022), the CD, FS, and XAS measurements described in the present study.

## Data Availability

The original contributions presented in the study are included in the article/Supplementary Material, further inquiries can be directed to the corresponding author.

## Footnotes

1 For the readers’ convenience, we report here the amino acid sequence of the mature, 81‐210, FXN. SGTLGHPGSL DETTYERLAE ETLDSLAEFF EDLADKPYTF EDYDVSFGSG VLTVKLGGDL GTYVINKQTP NKQIWLSSPS SGPKRYDWTG. KNWVYSHDGV SLHELLAAEL TKALKTKLDL SSLAYSGKDA. The α1-helix, from Glu92 to Leu113, is in red. The α2-helix, from Leu182 to Ala193, is in green. The β-sheet core, from Asp124 to Trp168 is in blue.

2 We may remark here that Co^2+^ has another property that is extremely useful for our XAS measurements: unlike Fe^2+^, it is a very resilient species under radiation damage.

3 This nomenclature is defined in the footnote in the introductory section.

4 The amino acid sequence of the mature, 81-210, wt-FXN is given in the footnote in the introductory section.

5 In this analysis, when two Tm are reported (see Table 2), the smallest is considered as already at this temperature unfolding is starting to take place.
