# Supplementary material for: Metal Ion Binding in Wild-Type and Mutated Frataxin: A Stability Study
Source: Front Mol Biosci. 2022 May 31;9:878017. doi: 10.3389/fmolb.2022.878017 (PMC9195147; doi:10.3389/fmolb.2022.878017)
Supplement: Supplementary file 1 [file DataSheet1.docx]

*Supplementary Material*

**Supplementary Figure 1. Far-UV CD spectra and thermal unfolding of hFXN isoforms.** Far‐UV CD spectra of hFXN 90-210 (A) and hFXN 81-210 (C) in the native conformation (solid line), before the thermal unfolding, and after the cooling phase (dashed line) at the end of the 20-95 thermal unfolding phase, were recorded in a 0.1‐cm quartz cuvette as described in Materials and methods. Thermal unfolding transitions (solid line) of hFXN 90-210 (B) and hFXN 81-210 (D); the dashed lines represent the profile obtained by cooling from 95 to 20°C, at the end of the 20-95 thermal unfolding phase. The molar ellipticity at 222 nm ([Θ_222_]) was monitored continuously every 0.5 °C.

**Supplementary Figure 2. Far-UV CD spectra and thermal unfolding of 90-210 hFXN variants.** Far‐UV CD spectra of D104G (A), Y123S (C) and S161I (E) in the native conformation (solid line), before the thermal unfolding, and after the cooling phase (dashed line) at the end of the 20-95 thermal unfolding phase, were recorded in a 0.1‐cm quartz cuvette as described in Materials and methods. Thermal unfolding transitions (solid line) of D104G(B), Y123S (D) and S161I (F); the dashed lines represent the profile obtained by cooling from 95 to 20°C, at the end of the 20-95 thermal unfolding phase. The molar ellipticity at 222 nm ([Θ_222_]) was monitored continuously every 0.5 °C

**Supplementary Figure 3. Far-UV CD spectra of 90-210 hFXN wild type and variants in the presence of Co^2+.^** Far‐UV CD spectra of hFXN 90-210 wild type (A), D104G (B), Y123S(C) and S161I (D) in the native conformation (solid line), before the thermal unfolding, and at the end of the cooling phase (dashed line) at the end of the 20-95 thermal unfolding phase, were recorded in a 0.1‐cm quartz cuvette in the presence of 0.8 Co^2+^ equivalents, as described in Materials and methods.
